# Smartphone survey data reveal the timecourse of changes in mood outcomes following vitamin C or kiwifruit intervention in adults with low vitamin C

**DOI:** 10.1017/S0007114523002787

**Published:** 2024-04-28

**Authors:** Benjamin D. Fletcher, Jillian J. Haszard, Margreet C. M. Vissers, Tamlin S. Conner

**Affiliations:** 1 Faculty of Medical and Health Sciences, University of Auckland, Auckland, New Zealand; 2 Biostatistics Centre, University of Otago, Dunedin, New Zealand; 3 Centre for Free Radical Research, Department of Pathology and Biomedical Science, University of Otago, Christchurch, New Zealand; 4 Department of Psychology, University of Otago, Dunedin, New Zealand

**Keywords:** Nutrition, Daily diary, Mental health, Flourishing, Well-being, Vitality, Diet

## Abstract

Vitamin C-rich foods can improve mood; however, the timecourse of these benefits is unknown. This study utilised intensive longitudinal smartphone surveys from a three-armed placebo-controlled trial to determine mood-related changes following supplementation with vitamin C (250 mg tablet/d), kiwifruit (2 SunGold™ kiwifruit/d) or a placebo (1 tablet/d). Secondary data were analysed from the KiwiC for Vitality trial (Trial ID: ACTRN12617001031358). Adults (*n* 155, 63 % female, aged 18–35 years) with low plasma vitamin C (<40 μmol/l) completed a 14-d lead-in, 28-d intervention and 14-d washout. Participants self-reported vitality (SF-36), mood (POMS total mood disturbance), flourishing (flourishing scale), sleep quality, sleep quantity and physical activity every second day using smartphone surveys. Plasma vitamin C, measured fortnightly, reached saturation after 2 weeks of vitamin C or kiwifruit supplementation. Kiwifruit supplementation improved vitality and mood within 4 days, peaking around 14–16 days, and improved flourishing from day 14. Vitamin C marginally improved mood until day 12. Incremental AUC analyses revealed significant overall effects of kiwifruit consumption on vitality and mood compared with placebo, which were stronger than effects for vitamin C tablets, but attenuated when adjusting for covariates. Sensitivity analyses of participants with low baseline vitamin C status revealed improved mood (vitamin C and kiwifruit) and flourishing (kiwifruit only). This is the first study to use intensive smartphone surveys to model the day-to-day timecourse of mood-related states following vitamin C intervention and highlights the value of using smartphone surveys to reveal the temporal changes in mood-related outcomes following nutrient supplementation.

A healthy diet rich in fruits and vegetables has been linked to better mental well-being^(1–5)^. Critically, diets rich in nutrient-dense fruits and vegetables may causally benefit mental health by lowering depressed mood or improving well-being^(6–10)^. Although the biochemical pathways linking fruits and vegetables to psychological symptoms are likely to be varied and complex^(11,12)^, one vitamin that may contribute to such changes is vitamin C. Vitamin C is involved in synthesising neurotransmitters, peptide hormones and acts as a catalyst for several enzymes, all of which are required to maintain and promote healthy brain functioning, including mood^(13)^.

A recent systematic review of vitamin C and neuropsychiatric impairment identified the need to investigate the effects of vitamin C replacement on psychological outcomes in people with vitamin C deficiency^(14)^. Vitamin C intake has been associated with improved mood, vitality, well-being and lower depression, while vitamin C deficiency is associated with higher depression and cognitive impairment^(14–16)^. Intervention studies have shown that supplementation with vitamin C or vitamin C-rich fruits, like kiwifruit, can improve mood and vitality in people with low baseline vitamin C levels^(17–19)^. Kiwifruit is an excellent source of vitamin C, which has shown a dose-dependent reduction in depression and total mood disturbance in young men who consume low levels of fruit and vegetables^(17)^. Previously, we demonstrated that two SunGold™ kiwifruit/d can promote markers of subjective vitality after 2 weeks of supplementation (KiwiC for Vitality study; Conner *et al*.^(18)^). Improvements were similar for participants supplemented with vitamin C, though the greatest improvements occurred in participants with low vitamin C levels at baseline (< 40 µmol/l).

However, limited research has assessed how quickly mood improvements occur after introducing vitamin C supplements or whole food sources. For example, it is unknown whether mood changes emerge within days or weeks of vitamin C supplementation. Most interventions rely on traditional designs in which patient-reported outcomes are measured at baseline and follow-up without attention to the intervening period. The present study aims to fill that gap by analysing smartphone survey data collected across an 8-week dietary intervention study^(18)^ to understand how daily vitality, mood and well-being changes between baseline and follow-up in adults recruited with low vitamin C levels.

Intensive smartphone surveys are also known as daily diary methods, experience sampling methods, ecological momentary assessment or ambulatory assessment and have become more common due to the ease, accessibility and affordability of smartphones^(20)^. These methods capture data in real time repeatedly over time and can help uncover the timecourse of changes in real-world environments^(21)^. For example, they can determine how quickly symptoms change over time, whether people improve steadily and incrementally, or if there is a non-linear or step-wise improvement suggesting a potential threshold process. Measuring micro-level timecourse changes is particularly important when assessing mood outcomes, which tend to fluctuate daily and are sensitive to biopsychosocial intervention^(22,23)^.

Although smartphone tools are becoming increasingly popular in nutrition research^(24,25)^, few nutrition interventions incorporate intensive smartphone surveys to measure day-to-day changes (for ecological momentary assessment applications in nutrition research, see Hand and Perzynski^(26)^, and Maugeri and Barchitta^(27)^). One nutritional intervention provided participants with fruits and vegetables, vouchers to purchase fruits and vegetables or a control group and measured daily mental well-being through smartphone surveys over 2 weeks^(10)^. Participants provided with fruits and vegetables reported increased vitality, flourishing and motivation after 2 weeks, while participants in the comparison groups reported no increase in well-being outcomes^(10)^. However, daily mood changes relative to baseline were not assessed to determine when benefits were observed. In another study, participants were supplemented with Berocca®, a multivitamin containing vitamin C (500 mg), or a placebo for 33 days^(28)^. Daily diary methods were used both morning and night at baseline and on days 7, 14, 21 and 28. Participants consuming Berocca® reported greater concentration, physical stamina and mental stamina in the evenings but not in the mornings relative to baseline. However, Berocca contains other ingredients, which limits conclusions specifically about vitamin C.

The timecourse of mood benefits from dietary vitamin C has yet to be determined, yet relatively fast action may be possible. Previously, large single doses of vitamin C have reduced fatigue and anxiety (10 g (intravenous) and 1 g (oral), respectively) within 2 h of administration^(29,30)^. Furthermore, acutely hospitalised patients with depleted vitamin C supplemented with vitamin C (1 g/d) reported reduced total mood disturbance assessed on average 8·7 days later, relative to patients receiving vitamin D tablets^(31)^. Moreover, the KiwiC for Vitality trial showed vitality improvements after 2 weeks of supplementation with kiwifruit or vitamin C tablets^(18)^. Thus, the timecourse may range from hours to 14 days. In the current study, we used data visualisations and mixed modelling to explore smartphone survey data from the KiwiC for Vitality trial to determine: (i) the timecourse of the changes (i.e. how many days into the intervention benefits may occur) and (ii) the overall or summary effect of the intervention on smartphone-assessed outcomes across the 28-d intervention. For the summary effects, two sensitivity analyses were conducted; one adjusted for confounding variables and one determined changes in outcomes for people with consistently low baseline vitamin C levels, who may benefit more from a vitamin C intervention than participants with saturated vitamin C levels.

## Materials and methods

### Study design and procedure

This paper reflects an exploratory analysis of secondary data from the KiwiC for Vitality trial. The KiwiC for Vitality trial was an 8-week, three-arm, parallel-group, placebo-controlled dietary intervention trial in participants with low vitamin C. The study was preregistered with the Australian and New Zealand Clinical Trial Registry (Trial ID: ACTRN12617001031358) and approved by the New Zealand Health and Disability Ethics Committee (17/NTB/104). Over 2 years, participants were recruited from tertiary education campuses in Dunedin, New Zealand (2017–2018). The trial consisted of a 2-week lead-in period, a 4-week intervention and a 2-week washout. For the 4-week intervention, participants were allocated to consume either a chewable placebo tablet, a chewable vitamin C tablet, or two SunGold® kiwifruit each day. For the full study protocol, CONSORT Diagram, CONSORT Checklist, inclusion-exclusion criteria, randomisation and primary results, refer to Conner *et al*.^(18)^ with further detail in Conner *et al.*(^32^). In brief, participants attended an initial screening session to provide a blood sample to determine if they were low in vitamin C (<40 µmol/l) and further eligibility (see *Participants* section). Participants enrolled in the study attended an in-person clinic every fortnight during the 8-week study (five visits in total). Fortnightly visits involved providing a fasting blood sample for vitamin C analysis and completing standard patient-reported outcomes retrospective measures of vitality, mood and well-being (reported in Conner *et al.* ^(18)^). Smartphone surveys were sent to participants every second day (see *Assessments – Smartphone Surveys* below).

### Participants

A total of 167 participants were allocated to a condition after being screened for low blood plasma vitamin C (<40 µmol/l), owning a smartphone, current student, aged 18–35 years, fruit intake, vegetable intake, medication, supplement use, excessive alcohol consumption, kiwifruit allergies, smoking status, diabetes, bleeding disorders and fainting due to fear of needles. Twelve participants had insufficient data for the exploratory analysis (less than 50 % of smartphone data during the study period). Thus, 155 participants were included in the current analysis. Although all participants initially had low blood plasma vitamin C levels (<40 µmol/l) at study entry (screening blood sample), due to the variable nature of vitamin C levels, some participants exhibited vitamin C levels above saturation levels (>60 µmol/l) during the lead-in period. Therefore, sensitivity analysis was conducted on 125 participants who had sufficient data and were consistently below saturation blood plasma vitamin C (<60 µmol/l) during the 2-week lead-in period and were likely to benefit most from the intervention.

### Randomisation

Group randomisation was employed to avoid participants being exposed to the other conditions (e.g. participants seeing others receiving kiwifruit when they received tablets or vice versa). Groups were based on scheduled clinic visits, either on separate days, or across days, to ensure that everyone attending the same clinic would receive either kiwifruit or tablets. Participants receiving tablets at a given clinic were typically a random mix of active and placebo participants. The allocation of clinics was determined by author TSC using a random number generator. Allocation to conditions occurred after participants were enrolled in their clinic day (usually Mon – Thurs). A delay in the delivery of tablets meant that clinics in the first and second waves of data collection were non-randomly allocated to the kiwifruit condition, and those in the third and fourth waves to the tablet conditions (randomised to placebo or vitamin C), with the remaining clinics randomised as intended (6 data collection waves in total). This introduced a non-randomised element into our study, as addressed elsewhere by Conner *et al*.^(18,32)^. The statistical analyses accounted for randomisation clusters for both waves of data collection and the day of the week participants attended clinic visits.

### Blinding

All research assistants and participants were double-blinded to tablet conditions. It was not possible to blind research assistants or participants to the kiwifruit condition. The separation of allocation clinics meant that participants were unaware of the nature of the other treatment conditions. Condition information was kept in an electronic password-protected document by TSC and unblinded following data collection and entry.

### Interventions

Participants in the active arms received an equivalent dose of 250 mg of vitamin C/d delivered via one chewable vitamin C tablet or two SunGold® kiwifruit. Participants in the placebo arm received a tablet matched for appearance and flavour with the vitamin C tablet but with no active vitamin C ingredients (both tablets manufactured by Tishcon Corporation). Tablets were bottled and labelled by TSC with the label including only the participant’s first and last name and tablet instructions to ‘chew one tablet daily, store in a dark, dry place and return the bottle and unused tablets on next visit’. Participants were given their tablets or a bag of fresh kiwifruit at the start of the intervention (day 0) and 2 weeks into the intervention (day 14). They were instructed to consume their tablets or kiwifruit any time each day over the 4-week intervention period.

### Assessments

#### Smartphone surveys

A survey URL link was sent to participants’ smartphones every second day throughout the 8-week study using an automated text message service (Message Media). This every-other-day schedule was selected to reduce participant burden while retaining as much temporal resolution as possible to detect time-related changes in mood outcomes^(33)^. Surveys began on the evening of the participants’ first clinic visit and ended on the evening of their last clinic visit, resulting in twenty-nine surveys. Surveys were delivered every other day between 5:45 pm and 6:15 pm. Survey links were available to be completed any time during the evening until 2 am. The time surveys were sent to participants varied, utilising a semi-random schedule (within the fixed window for delivery) to avoid participants adapting and predicting the survey delivery time, which can result in disengagement. Any participants who missed a survey were sent an automated email prompt to complete a survey the following evening.

Smartphone texts contained the survey URL and a short message corresponding to the lead-in, intervention or washout periods. The lead-in period messages included a thank-you for participating, a reminder of payment incentives, email contact information for queries or issues, a reminder to complete diet records, a reminder to refrain from drinking fruit juice and clinic visit reminders. The intervention period messages were similar to the lead-in period, although they also included a reminder to consume their supplements or kiwifruit. The washout period consisted of the same messages as the lead-in period. If participants missed a smartphone survey, they were sent an automated email the following evening, including a link to complete a make-up survey. Participants who missed two consecutive surveys were sent an additional text reminding them of the incentive that each survey was worth $2 and added towards a $100 completion bonus for completing all aspects of the study. If another consecutive survey was missed, author (BDF) contacted the participant to confirm no technical issues and ensure that the participant was still happy to participate through email, text and phone call prompts.

Participants were remunerated $2 per survey response (maximum of $58), as well as $20 for each fortnightly clinic visit (maximum of $100), $10 for each dietary record completed (maximum of $30) and incentivised with a proportion of a $100 completion bonus for completing aspects of the study (i.e. $100 for all twenty-nine smartphone surveys, five clinic visits and three diet records).

#### Measures

The smartphone survey contained seventeen items, consisting of the following measures, in this order:

##### The rand 36-item short form fatigue-energy subscale

The smartphone survey included the SF-36 Fatigue-Energy Subscale^(34)^, a validated four-item measure of energy-fatigue (or vitality). The four items were *Today… Did you feel full of life? Did you have a lot of energy? Did you feel worn out? Did you feel tired?* (The item *‘full of life*’ was modified from the original ‘*full of pep*’ to update the wording for the study population). Each item was rated on a six-point Likert-type scale from 0 (none of the time) to 5 (all of the time). Responses were recoded to 0 to 100 in twenty-point intervals per the SF-36 guidelines. Two of the items, *worn out* and *tired*, were reversed scored. All four -items were averaged, resulting in a maximum score of 100, which indicates higher vitality and lower fatigue (within-person *α* = 0·569, calculated for nested data as recommended by Nezlek^(35)^.

##### Profile of mood states – 18-item short form

The smartphone survey included six rotating mood items from a wider bank of eighteen items from the POMS short form (POMS-SF)^(36)^. Items were rated for how people felt ‘today’ on a five-point Likert-type scale ranging from 0 (‘not at all’) to 4 (‘extremely’). The wider bank of eighteen mood items consisted of three mood items for each of the six mood subscales (anxiety-tension, depression-dejection, anger-hostility, vitality-activity, fatigue-inertia and confusion-bewilderment). The selected items from the POMS-SF varied in each survey. One item from each of the six factors was randomly included to keep assessments as short as possible and prevent participants from forming habitual answering patterns over the 8-week trial. Thus, POMS subscales were not calculated as participants only answered six POMS items for every smartphone survey. If one of the randomly presented items from within each subscale was missing (e.g. missed by a participant), day-specific individual mean imputation was conducted using that person’s responses to POMS items on the day. If more than one item was missing, imputation was not conducted (overall, 12 % of POMS items were missing (including surveys not started) and 4·5 % were mean imputed). The six items were rescaled (multiplied by 5) to retain the original scale scoring and summed (reverse scoring the *vitality-activity* item) to calculate a total mood disturbance score for each survey day. Total mood disturbance ranged from −20 to 100, with higher values indicating greater mood disturbance and worse mood (within-person *α* = 0·110).

##### Shortened daily flourishing scale

The smartphone survey included a short four-item flourishing scale that assesses positive psychological well-being in reference to feelings on that day. The four items were *Today, I was engaged and interested in my daily activities. Today, I led a purposeful and meaningful life. Today, I was a good person and lived a good life. Today, I was competent and capable in my important activities.* These four items were selected from the original eight-item flourishing scale^(37)^ and adapted for a daily format by changing the timeframe to today. Each item was rated on a seven-point Likert-type scale ranging from 1 (strongly disagree) to 7 (strongly agree). A short three-item version of the flourishing scale was previously shown to have good validity^(10)^; in the current study, we used the same three items plus added the ‘important activities’ question because it contributed to good within-person reliability in a prior dataset. The four items were averaged (within-person *α* = 0·867) to give an overall daily flourishing score ranging from 1 to 7, with higher scores indicating greater flourishing.

##### Sleep quality, sleep quantity and physical activity

The end of the smartphone survey included self-reported sleep quantity, sleep quality and physical activity measures as covariates in the main analyses. Sleep quantity was measured using the question, ‘*Approximately how many hours did you sleep last night?’.* Participants answered using a pulldown menu that ranged from 0 to 24 h, increasing in increments of 30 min. Sleep quality was assessed using the validated Sleep Quality-Numeric Rating Scale, which is a single question rated on a 10-point Likert scale: ‘*Please rate the quality of your sleep last night’: 0 (‘worst possible sleep’) to 10 (‘best possible sleep’)* ^(38,39)^. Physical activity was assessed using the single-item activity questionnaire: ‘*Today, have you done a total of 30 min or more of physical activity, enough to raise your breathing rate? (sport, exercise, brisk walking or cycling)’*, which participants responded to using *No* or *Yes*. The physical activity question was adapted from a validated single-item questionnaire to assess physical activity by changing instructions to ‘*today*’ in accordance with smartphone methods rather than ‘*in the past week/past month*’^(40)^.

#### Data preparation and statistical analysis

During the study, some participants completed surveys on consecutive days, while others completed a make-up survey the day after survey distribution. Thus, participant data were binned into 2-d intervals (i.e. surveys completed on Day 0 or Day 1 were binned into Day 0, surveys completed on Day 2 or Day 3 were binned into Day 2), to keep analyses consistent with the temporal resolution of the distribution of daily diary surveys.

All statistical analyses and visualisations were undertaken in Stata 17.0 (StataCorp.). Statistical significance was defined as *P* < 0·05.

For the timecourse investigation, we visualised the changes over time using two different figures for each outcome (vitality, mood and flourishing). The first figure simply plotted the mean change in the outcome from baseline every second day, with error bars as standard errors of the mean. This was carried out for all three groups (placebo, Vitamin C and kiwifruit) and included the washout period. This figure visually demonstrated when the biggest improvements were and if they were sustained. The second figure plotted the *effects* of the interventions (Vitamin C and kiwifruit compared with placebo) as mean differences from placebo and 95 % CI on every second day throughout the intervention (and washout period). These effects were estimated from mixed effects regression models for each day, where the outcome variable was the dependent variable, the intervention groups were the independent variable (with placebo as the reference group) and adjusted for baseline vitality, mood, or flourishing. There were two randomisation cluster variables that were included as random effects, with the second cluster nested within the first. The first cluster variable identified the week of the trial (there were 6 weeks in total), and the second cluster variable identified clusters of randomisation within the week (e.g. if group-randomised by day of the week). Care should be taken not to use ‘statistical significance’ for the interpretation of these graphs where many timepoints are presented, but rather to interpret the *pattern* of results to help understand the timecourse^(41)^.

For the summary effects over 28-d, incremental AUC (iAUC) was used as a summary measure of vitality, mood and flourishing changes for each participant across the 28-d intervention period. This is a measure of response to intervention with time–series data by plotting outcome measures against time and using cubic splines to estimate the area under the curve from baseline^(42)^. To help with the interpretation of iAUC effect sizes, iAUC was also standardised and presented in units of sd with ∼0·3 SD suggesting a small effect, 0·5 SD a moderate effect and >0·8 SD a large effect^(43)^. To assess the differences in iAUC between intervention groups, mixed effects regression models were used with iAUC as the dependent variable and randomised group as the independent variable. Random effects were also used to account for randomisation clusters as before. Sensitivity analyses were then carried out, first, with adjustment for mean sleep quality and physical activity over the intervention, age and ethnicity, and second, by testing participants with below saturation baseline vitamin C levels (<60 µmol/l) during the lead-in period. Differences in iAUC between intervention conditions during the 14-d washout period were also assessed. Residuals of all models were plotted and visually assessed for homoskedasticity and normality.

## Results

### Descriptive statistics

A total of 167 participants were allocated to a condition and completed the baseline measures; however, twelve participants had more than 50 % missing data for their smartphone data during the lead-in or intervention periods. As such, 155 participants with sufficient data were included in the secondary analysis. Table 1 presents the participant characteristics of the analysed sample.

**Table 1.** Demographic and baseline characteristics of the analysed sample (*n* 155)

	Placebo group		Vitamin C group		Kiwifruit group	
	*n*	%	*n*	%	*n*	%
*n*	50		51		54	
Gender*						
Male	17	34·0	19	37·3	20	37·0
Female	33	66·0	32	62·7	33	61·1
Ethnicity						
Caucasian	17	34·0	17	33·3	25	46·3
Asian	23	46·0	25	49·0	16	29·6
Māori & Pacific	2	4·0	1	2·0	4	7·4
Other	8	16·0	8	15·7	9	16·7
Low vitamin C during baseline†	44	88·0	40	78·4	41	75·9

	Mean	sd	Mean	sd	Mean	sd
Age, years	22·1	3·4	22·9	4·5	20·4	1·9
BMI, kg/m^2^	23·7	5·8	23·6	3·7	24·1	3·6
Baseline‡ vitality	60·9	14·0	64·0	16·2	60·9	14·1
Baseline‡ total mood disturbance	5·6	11·6	5·2	15·2	6·8	11·7
Baseline‡ flourishing	5·0	0·9	5·1	1·1	4·9	0·8
Mean sleep quality§	6·5	1·2	6·8	1·1	6·7	1·0
Mean sleep quantity (hours)‖	7·1	0·7	7·2	0·7	7·2	0·7
Proportion of days with physical activity	0·4	0·3	0·5	0·3	0·4	0·3
Number of smartphone surveys completed (out of 29)	27·4	2·1	26·9	2·9	26·5	2·9
Range of smartphone surveys completed	21–29		15–29		18–29	

BMI = body mass index; sd = standard deviation.

* One participant identified as gender diverse from the Kiwifruit group.

† Participants maintained below saturation levels of vitamin C (<60 µmol/l) during the lead-in period.

‡ Baseline scores calculated as the mean from all days in the 2-week lead-in phase. Vitality was calculated as the sum score of the Rand 36-Item Short Form (SF-36) Fatigue-Energy Subscale (scale range 0 to 100; higher scores indicate greater vitality). Total Mood Disturbance was measured using selected items from the Profile of Mood States (scale range was −20 to 100; higher scores indicate poorer mood). Flourishing was measured using the shortened daily flourishing scale (scale range was 1 to 7; higher scores indicate better well-being).

§ Sleep quality-numeric rating scale (SQ-NRS) measured every second day on a scale from 0 (‘worst possible sleep’) to 10 (‘best possible sleep’).

‖ Sleep quantity was measured using a single item, ‘*Approximately how many hours did you sleep last night?*’ every second day on a scale from 0 to 24 h, increasing in increments of 30 min.

Note: Twelve participants were removed because they had too much missing smartphone survey data (>50 % missing over the intervention period or no baseline data). This accounts for *n* 155 compared with the original dataset with *n* 167.

The smartphone survey link was available every evening during the study period, resulting in some participants completing surveys on consecutive days; these data were averaged within each 2-d bin and accounted for 2·5 % of responses. Based on binned survey data, compliance was high at 92·8 %, with 4170 out of 4495 possible smartphone surveys during the study period being answered (26·9/29 mean surveys completed: range 15 to 29) for the exploratory analysis sample (*n* 155). There was no difference in compliance between conditions for the full sample (*F* (2164) = 0·336, *P =* 0·715; *n* 167) or exploratory sample analysed (*F* (2152) = 0·1·77, *P =* 0·174; *n* 155).

### Timecourse investigation

**Fig. 1.**
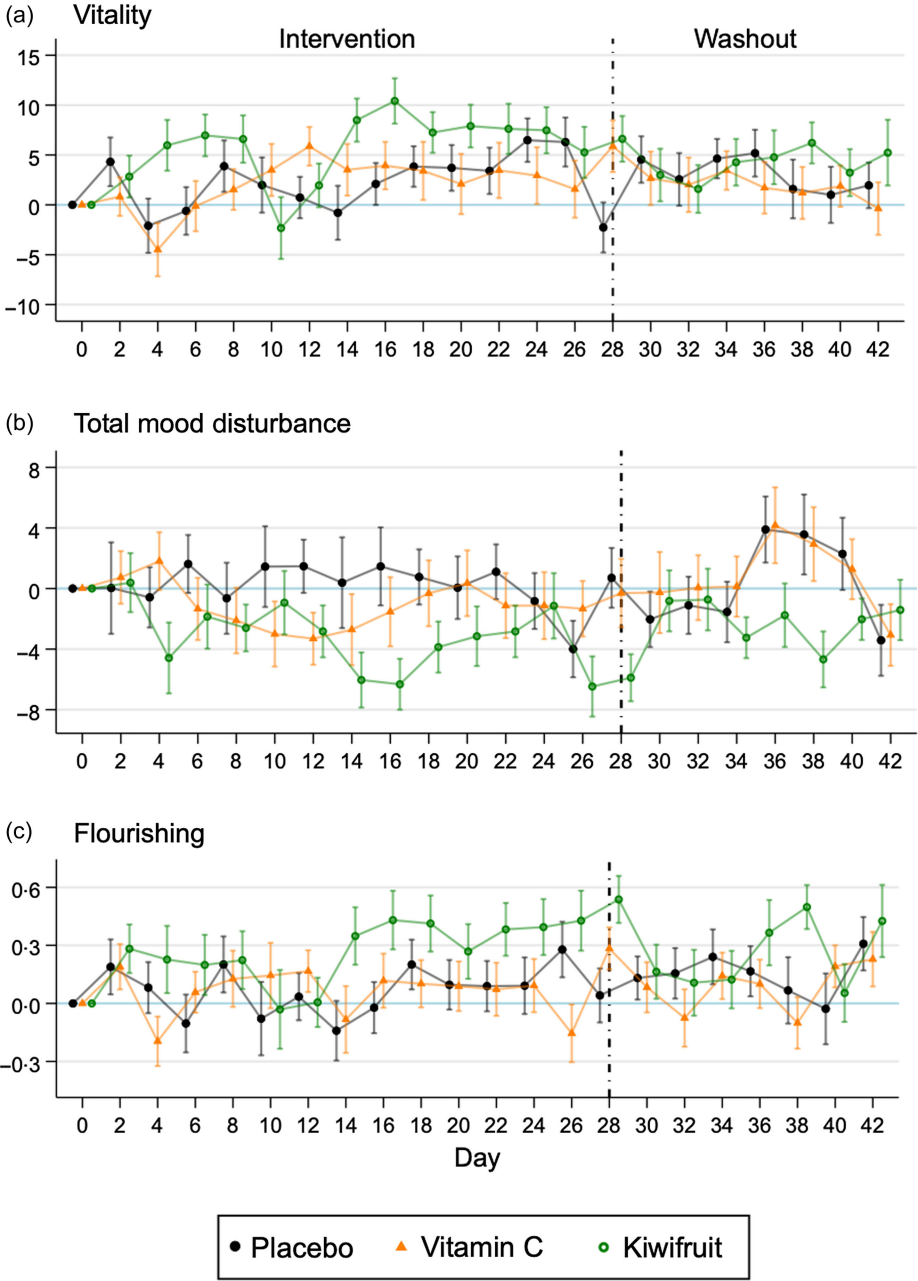
Mean (se) difference in scores from baseline for vitality, total mood disturbance and flourishing (*n* 155) by randomised group across the intervention period (28-d) and the washout period (14-d).

Fig. 1 shows the timecourse of changes in outcomes over time for the three groups. Vitality improvements were seen to occur within 4-d of the kiwifruit intervention, which dropped on day 10 and then peaked rapidly on day 16 (Fig. 1(a)). However, there was a large amount of variability in daily vitality scores for all groups, and improvements were not consistent over time (Fig. 1(a)). Washout vitality scores were similar across the three groups. Total mood disturbance showed a similar improvement with the kiwifruit intervention emerging around 4-d, which peaked at day 16, while the Vitamin C group showed a smaller improvement peaking at day 12 that returned to baseline thereafter (Fig. 1(b)). Flourishing showed improvement in the kiwifruit group later in the intervention – starting at day 14 – with a sustained effect through to the end of the 28-d intervention (Fig. 1(c)). Improvement in flourishing was not apparent in the other groups.

**Fig. 2.**
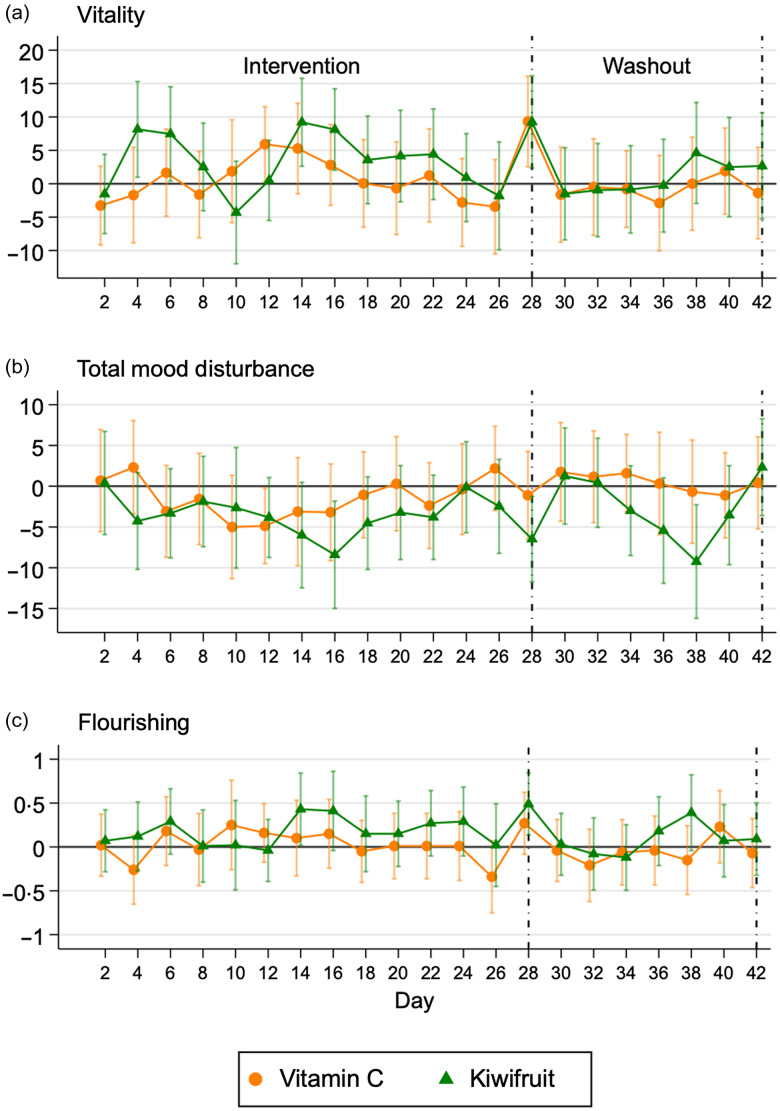
Mean differences (95 % CI) in vitality, total mood disturbance and flourishing by day for vitamin C and kiwifruit groups compared with the placebo. Adjusted for baseline and clustered randomisation. (*n* 155).

Fig. 2 shows the timecourse of the *effects* of the two active intervention arms (Vitamin C and kiwifruit groups) compared with the placebo group after adjustment for baseline and accounting for randomisation clusters. Here, the kiwifruit intervention was superior to the Vitamin C intervention. Effects of kiwifruit on vitality and total mood disturbance were observed early on – within the first 4-d – and peaked around days 14–16 (Fig. 2(a) and (b)). However, these effects dissipated in the third week of intervention. Small effects of kiwifruit supplementation on flourishing were seen after 2 weeks of intervention but were brief and not sustained relative to the placebo group.

### Summary effects over 28 days

Table 2 presents the iAUC results. Overall, there was no statistically significant effect of Vitamin C supplementation compared with placebo across the 28-d of intervention for vitality (Standardised iAUC = −0·08 (−0·46, 0·30)), Total mood disturbance (Standardised iAUC = −0·23 (−0·61, 0·15)), or flourishing (standardised iAUC = −0·06 (−0·44, 0·32)), when adjusting for baseline and accounting for randomisation clusters. However, kiwifruit supplementation showed a statistically significant overall increase in vitality (standardised iAUC = 0·39 (0·01, 0·77)) and a decrease in total mood disturbance (standardised iAUC = −0·43 (−0·85, −0·01)) across the 28-d, relative to placebo. When comparing kiwifruit to Vitamin C supplementation, there was a significant increase in flourishing for kiwifruit compared with Vitamin C (standardised iAUC = 0·50 (0·09, 0·91)).

**Table 2.** The summary effects of vitamin C and kiwifruit on overall vitality, total mood disturbance, and flourishing across 28 days of intervention (*n* 155)

	Placebo (*n* 50)		Vitamin C (*n* 51)		Kiwifruit (*n* 54)		Effect of vitamin C compared to placebo		Effect of kiwifruit compared to placebo		Effect of kiwifruit compared to vitamin C	
	Mean	sd	Mean	sd	Mean	sd	Mean difference	95 % CI*	Mean difference	95 % CI*	Mean difference	95 % CI*
Vitality												
iAUC†	50	251	28	321	161	233	−23	−126, 81	**108**	2, 62	77	−112, 265
Standardised iAUC†	−0·11	0·91	−0·20	1·17	0·29	0·85	−0·08	−0·46, 0·30	**0·39**	**0·01, 0·77**	0·28	−0·56, 0·40
Total mood disturbance												
iAUC†	3	244	−48	275	−94	174	−54	−144, 35	−**102**	−**201, −3**	−50	−174, 74
Standardised iAUC†	0·22	1·04	0·00	1·17	−0·20	0·74	−0·23	−0·61, 0·15	−**0·43**	−**0·85, −0·01**	−0·21	−0·74, 0·31
Flourishing												
iAUC†	1·1	17·3	−0·4	15·4	7·3	13·5	−1·0	−7·0, 5·0	6·6	−0·2, 13·4	**7·9**	**1·4, 14·3**
Standardised iAUC†	−0·11	1·10	−0·20	0·98	0·29	0·86	−0·06	−0·44, 0·32	0·42	−0·01, 0·86	**0·50**	**0·09, 0·91**

* Mean difference (95 % CI) estimated using mixed effects regression models with two levels of clustering included as random effects.

† iAUC: net incremental area under the curve of scores across 28-d of intervention, with baseline taken as the mean of all scores in the 2-week lead-in.

Table 3 presents the sensitivity analyses of the iAUC results when adjusting for confounders (top) and when testing participants with low baseline vitamin C (bottom). Adjustment for sleep quality, physical activity, age and ethnicity did not change the otherwise null effects of the Vitamin C supplementation compared with Placebo across the 28-d; however, adjustment for confounders attenuated the summary effect of the kiwifruit supplementation on vitality and total mood disturbance across the 28-d compared with placebo (effects no longer significant). After adjustment for potential confounders, the benefit of kiwifruit compared with Vitamin C supplementation on flourishing was strengthened (standardised iAUC = 0·63 (0·19. 1·07), and the effect on vitality became statistically significant (standardised iAUC = 0·55 (0·17, 0·94)). As shown in Table 3 (bottom), the intervention effects were significant for total mood disturbance and flourishing among participants with low baseline vitamin C. Vitamin C supplementation showed significant improvements in total mood disturbance across the 28-d relative to placebo (standardised iAUC = −0·47 (−0·89, −0·04)). Kiwifruit supplementation showed a significant reduction in total mood disturbance (standardised iAUC = −0·52 (−1·04, −0·01)) and a significant increase in Flourishing (standardised iAUC = 0·52 (0·07, 0·96)) across the 28-d relative to placebo. Kiwifruit supplementation showed a greater benefit than Vitamin C supplementation on flourishing (standardised iAUC = 0·45 (0·03, 0·88)). Table 4 shows that there were no significant effects of supplementation on vitality, mood disturbance or flourishing in the 2 weeks after supplementation ceased.

**Table 3.** Sensitivity analyses of the summary effects of vitamin C and kiwifruit on overall vitality, total mood disturbance, and flourishing across 28 days of intervention: adjustment for confounders (top) and effects in those with low vitamin C at baseline (bottom)

	*n*	Effect of vitamin C compared to placebo		Effect of kiwifruit compared to placebo		Effect of kiwifruit compared to vitamin C	
		Mean difference	95 % CI*	Mean difference	95 % CI*	Mean difference	95 % CI*
Adjustment for confounders†							
Vitality							
iAUC‡	155	−30	−1313, 72	102	−4, 209	**152**	46, 63
Standardised iAUC‡	155	−0·11	−0·48, 0·26	0·37	−0·02, 0·76	**0·55**	**0·17, 0·94**
Total mood disturbance							
iAUC‡	155	−50	−139, 40	−88	−192, 17	−55	−149, 38
Standardised iAUC‡	155	−0·21	−0·59, 0·17	−0·37	−0·82, 0·07	−0·24	−0·63, 0·16
Flourishing							
iAUC‡	155	−1·2	−7·2, 4·8	6·4	−0·6, 13·4	**9·9**	**3·0, 16·8**
Standardised iAUC‡	155	−0·07	−0·46, 0·31	0·41	−0·04, 0·85	**0·63**	**0·19, 1·07**
Low baseline vitamin C§							
Vitality							
iAUC‡	125	34	−78, 146	99	−18, 215	32	−153, 217
Standardised iAUC‡	125	0·12	−0·28, 0·53	0·36	−0·06, 0·78	0·12	−0·56, 0·63
Total mood disturbance							
iAUC‡	125	−**110**	−**211, −9**	−**123**	−**244, −3**	−27	−150, 95
Standardised iAUC‡	125	−**0·47**	−**0·89, −0·04**	−**0·52**	−**1·04, −0·01**	−0·12	−0·64, 0·40
Flourishing							
iAUC‡	125	1·0	−5·2, 7·3	**8·1**	**1·1, 15·1**	**7·1**	**0·5, 13·8**
Standardised iAUC‡	125	0·07	−0·33, 0·46	**0·52**	**0·07, 0·96**	**0·45**	**0·03, 0·88**

* Mean difference (95 % CI) estimated using mixed effects regression models with two levels of clustering included as random effects.

† Adjustment was made for average sleep quality across the intervention period, average physical activity across the intervention period, age and ethnicity.

‡ iAUC: net incremental area under the curve of scores across 28-d of intervention, with baseline taken as the mean of all scores in the 2-week lead-in.

§ Participants maintained below saturation levels of vitamin C (<60 µmol/l) during the lead-in period.

**Table 4.** The summary effects of vitamin C and kiwifruit on overall vitality, total mood disturbance, and flourishing across 14 days of washout after 28 days of intervention (*n* 148)

	Placebo (*n* 48)		Vitamin C (*n* 49)		Kiwifruit (*n* 51)		Effect of vitamin C follow-up compared to placebo follow-up		Effect of kiwifruit follow-up compared to placebo follow-up		Effect of kiwifruit follow-up compared to vitamin C follow-up	
	Mean	SD	Mean	SD	Mean	SD	Mean difference	95 % CI*	Mean difference	95 % CI*	Mean difference	95 % CI*
Vitality												
iAUC†	41	150	8	162	−2	163	−31	−93, 30	−40	−109, 28	−9	−72, 54
Standardised iAUC†	0·16	0·94	−0·05	1·02	−0·11	1·03	−0·20	−0·59, 0·19	−0·26	−0·69, 0·18	−0·06	−0·46, 0·34
Total mood disturbance												
iAUC†	−3	120	8	163	−45	103	10	−41, 62	−48	−105, 8	−59	−128, 11
Standardised iAUC†	0·08	0·91	0·17	1·23	−0·24	0·78	0·08	−0·31, 0·47	−0·36	−0·79, 0·07	−0·44	−0·97, 0·08
Flourishing												
iAUC†	1·5	9·8	−0·8	8·8	−1·2	10·0	−2·3	−6·1, 1·4	−2·8	−6·5, 1·0	−0·3	−4·1, 3·5
Standardised iAUC†	0·18	1·03	−0·07	0·92	−0·11	1·04	−0·24	−0·64, 0·15	−0·29	−0·68, 0·10	−0·03	−0·43, 0·37

* Mean difference (95 % CI) estimated using mixed effects regression models with two levels of clustering included as random effects.

† iAUC: net incremental area under the curve of scores across 14-d of washout, with baseline taken as the mean of all scores in the 2-week lead-in.

## Discussion

The current smartphone three-armed placebo-controlled trial used fine-grained temporal resolution to provide novel insight into day-to-day changes in mood-related outcomes following vitamin C or kiwifruit supplementation in otherwise healthy adults with low vitamin C levels. Both vitality and mood improved within 4-d of supplementation with two SunGold® kiwifruit/d and peaked around 16-d of supplementation. Flourishing improved after 2 weeks of kiwifruit supplementation. However, vitality and mood improvements were not consistent and fluctuated from day-to-day. Interestingly, the timecourse of benefits was similar between outcomes, with participants in the kiwifruit condition showing initial improvements in 4-d but more consistent improvements after 2 weeks. This may suggest that nutritional interventions or small dietary changes could emerge quickly but may require 2 weeks to begin seeing more substantive mood improvements.

Previous research has assessed how vitamin C or multivitamin supplements may influence mental health outcomes over short time frames. For example, participants with low vitamin C levels given a single 10 g intravenous vitamin C dose reported decreased fatigue 2 h following administration^(29)^. Likewise, a 1000 mg oral dose reduced anxiety within 2 h for post-graduate students with high anxiety levels^(30)^. A vitamin C-enriched Berocca® study that utilised daily diaries showed that improvements in concentration, alertness and mental and physical stamina were prominent in the evening but not in the morning^(28)^. It may be that the mode of action of supplementation is relatively short, with supplements taken during the day having an effect in the evening but wearing off by morning. However, the time of day for supplementation was not specified in the current study, so we are not able to determine if the improvements from kiwifruit supplementation are due to a longer mode of action or if it is linked to the time-of-day participants consumed kiwifruit or vitamin C tablets. Future studies may need to specify scheduled supplement use to identify time-linked effects more accurately. The current study indicates that a whole-food source of vitamin C may have moderately fast-acting effects in as few as 4-d, with sustainable day-to-day mood-related benefits accumulating in up to 14-d.

We also examined the intervention effects observed across the entire 28-d period using the iAUC analyses. These analyses tested the overall intervention effect on the sum total amount of change across the 28-d of intervention. Unadjusted models showed significant overall effects of the kiwifruit intervention on vitality and total mood disturbance relative to placebo. However, when adjusting for confounding variables, the results were attenuated and no longer statistically different. This attenuation may be driven by differences between conditions or sample heterogeneity. There were differences in sleep quality and physical activity between conditions, with relatively lower sleep quality in the placebo group and greater physical activity in the vitamin C group, which are related to mood outcomes. There were also ethnic differences between conditions. The placebo and vitamin C conditions consisted of almost 50 % Asian participants, whereas the kiwifruit condition consisted of approximately 30 % Asian participants. It may be possible that there are cultural differences in how participants responded to questionnaire items, dietary differences or there may be genetic differences in vitamin C absorption or processing. Generally, the POMS, SF-36 and flourishing scales are well-validated, widely used across various populations and cultures and considered appropriate. However, there may be ethnicity-linked differences in the relationship between vitamin C and mood-related outcomes. For example, vegetarian diets and higher vitamin C status have been associated with poorer mental health for some Asian sub-groups^(44,45)^. Interestingly, Asian populations tend to have lower vitamin C levels than other ethnic groups, which is thought to be driven by food preparation (cooking technique and duration reduce vitamin C content), cultural factors, socio-economic status or genetic factors^(46–49)^. However, whether these factors may be related to vitamin C-mediated mental health outcomes is unknown. Further randomised controlled trials could help assess any potential impact of dietary interventions on mental health outcomes in general and by ethnicity.

Sensitivity analysis suggested that improvements in total mood disturbance may be greatest for participants with low vitamin C levels at baseline when supplemented with either vitamin C tablets or kiwifruit. Sensitivity analysis was consistent with previous findings, in which participants with consistently low vitamin C at baseline are more likely to benefit from nutritional intervention^(17,18)^. As expected, participants with the greatest potential for improvement reported greater mood benefits from the intervention. Mood improvements appeared to be stronger and occurred earlier for participants who consumed two SunGold® kiwifruit/d. Despite comparable vitamin C bioavailability from synthetic and whole food sources^(50,51)^, this study highlights that whole food sources may provide additional benefits for mental health than vitamin C alone in tablet form.

It is likely that other dietary compounds found in the whole food source (kiwifruit) also contributed to mental health improvements. In addition to high vitamin C content, SunGold® kiwifruit is a nutrient-dense fruit that is relatively high in fibre, folate (vitamin B_9_) and potassium, all of which have been linked to mental health outcomes^(52,53)^. For example, dietary fibre in kiwifruit may promote gut health. There is growing evidence that a healthy gut microbiome is associated with improved mood and mental health outcomes. Notably, higher fibre and folate have been associated with lower levels of depression^(54,55)^. Furthermore, potassium has been associated with increased vigour and lower levels of depression, tension and POMS total mood disturbance^(56)^. Kiwifruit also contain phytonutrients, such as flavonoids and carotenoids, which are associated with reduced oxidative stress and inflammation^(53)^. Anti-inflammatory and antioxidant pathways have been suggested to play a role in mood and mental health outcomes, such as depression and anxiety^(57,58)^. In addition to vitamin C, the antioxidant activity of kiwifruit may be driven by two bioavailable forms of Vitamin E (*α*-tocopherol and *δ*-tocomonoenol)^(53,59)^. However, the effect of vitamin E on mental health is currently inconclusive^(60)^. As kiwifruit contains a combination of nutrients, there is likely to be a synergistic nutrient interaction, which may have a more significant impact on mood compared with a single nutrient in isolation. While this study offers insights into the potential benefits of kiwifruit, further research is required to determine how other micronutrients may impact mental health in isolation or in varying combinations.

The current study had numerous strengths as the first controlled trial to examine the day-to-day changes in mood-related outcomes in response to a vitamin C or kiwifruit intervention. First, using a double-blind placebo-controlled arm allowed us to determine the relative effectiveness of the active micronutrient, vitamin C, in isolation or in comparison to a whole food over time. Second, the 8-week trial design with multiple baseline, intervention and washout time points allowed us to assess trajectories over time. The length of the 4-week intervention provided sufficient time to observe mood improvements. Additionally, extensive screening and strict inclusion and exclusion criteria ensured the intervention was targeted towards relatively healthy but low vitamin C-consuming individuals most likely to benefit from a dietary intervention. Finally, there was high compliance with the smartphone survey methodologies to track outcomes on a day-to-day basis allowing for more accurate assessments while minimising retrospective recall. The greater temporal resolution provided by the smartphone surveys allowed the results to reflect mood fluctuations in real-time outside of the laboratory setting.

A strength of the current study was testing each intervention day separately against baseline to determine when the mood benefits appear and the inclusion of smartphone tracking during a washout period to visualise the lasting effects of the intervention. It appears that mood-related benefits were rapidly depleted following the end of the intervention. Two days after the intervention, no benefits were observed for any condition. Whereas mood-related benefits took half a week to 2 weeks to accumulate (for either vitamin C or kiwifruit supplementation), their benefits were diminished rapidly following the end of the intervention. Thus, it is likely that consistent daily consumption of vitamin C-rich fruit or supplementation is required to maintain mood-related improvements.

The current study also had limitations. The methodology was limited by using a restricted number of items from the POMS-SF in an effort to reduce participant burden. Using a pool of items from the POMS-SF likely reduced reliability, and therefore these results should be interpreted with caution. However, it should be noted that the criteria for nested reliability of within-person variability is more relaxed than traditional reliability estimates^(35)^, and other measures used in the current study showed acceptable reliability. Additionally, this study was limited by data collection being every second day. There is no gold standard for daily diary methodologies; thus, researchers must use discretion when determining the length, content, frequency and delivery of assessments to manage participant burden and research outcomes. As the smartphone assessments were a secondary outcome, questionnaire links were only administered every second day to reduce participant burden. As such, some participants completed surveys on two consecutive days if they forgot they had completed a survey the night before or missed a survey entirely. Daily surveys would have circumvented this, although it may have resulted in a greater amount of missing data due to greater participant burden decreasing compliance. Future daily diary or ecological momentary assessment studies may be able to include daily mood measures to get greater temporal resolution of mood fluctuations with validated measures. In addition, participants were relatively psychologically healthy as a sample, with low baseline mood disturbance, which limited the potential for improvement. We speculate that the length and complexity of the study during the academic term might have deterred more distressed people from participating, or that such individuals are less motivated to volunteer for recruitment for an extended time intervention study. It is possible that benefits may have been stronger had we targeted recruitment towards a more distressed sample. Nevertheless, these exploratory analyses give a novel insight into the rate at which one may expect to benefit from kiwifruit or vitamin C supplementation in otherwise healthy adults 18 to 35 years old who present with low vitamin C levels.

It should also be noted that there appeared to be a spike in mood responses on days 14 and 28 of the intervention, which corresponded with clinic visits. It is possible that the face-to-face interactions with researchers made the study more salient in the participants’ minds and resulted in a positive response bias. Alternatively, participants received free supplements or kiwifruit (day 14) and were paid for their involvement at clinic visits, which may have boosted participants’ mood. However, the lack of similar spikes in the placebo condition or during the lead-in (days −14 and 0; Day 0 being the day participants received tablets or kiwifruit) and at washout (day 42) suggests that response bias, free supplements or payment were not involved in how participants responded to mood measures.

### Conclusion

Our study suggests that kiwifruit consumption, and to a lesser extent vitamin C supplementation, can lead to mood-related improvements in a relatively short period of time (from 4 to 16 days), even in a relatively healthy population with good mental health. Whole food sources (kiwifruit) showed greater and more consistent improvements over time than vitamin C alone, and improvements were more notable for individuals who had consistently low vitamin C levels at baseline. Future research should consider vitamin C as one possible mechanism that may contribute to mood improvements from fruit and vegetable consumption. Future nutrition research should also consider the temporal dynamics of mood change following dietary interventions using innovative smartphone technology.
